# Ergogenic Effects of Acute Caffeine Intake on Muscular Endurance and Muscular Strength in Women: A Meta-Analysis

**DOI:** 10.3390/ijerph18115773

**Published:** 2021-05-27

**Authors:** Jozo Grgic, Juan Del Coso

**Affiliations:** 1 Institute for Health and Sport, Victoria University, Melbourne, VIC 8001, Australia; 2 Centre for Sport Studies, Rey Juan Carlos University, 43959 Fuenlabrada, Spain; juan.delcoso@urjc.es

**Keywords:** ergogenic aid, supplements, resistance exercise

## Abstract

This meta-analysis aimed to explore the effects of caffeine ingestion on muscular endurance and muscular strength in women. Five databases were searched to find relevant studies. A random-effects meta-analysis of standardized mean differences (SMD) was performed for data analysis. Subgroup meta-analyses explored the effects of caffeine on upper-body and lower-body muscular endurance and muscular strength. Eight crossover placebo-controlled studies were included in the review. In the main meta-analysis that considered data from all included studies, there was a significant ergogenic effect of caffeine on muscular endurance (SMD = 0.25; *p* = 0.027) and muscular strength (SMD = 0.18; *p* < 0.001). In a subgroup analysis that considered only upper-body exercises, there was a significant ergogenic effect of caffeine on muscular endurance (SMD = 0.20; *p* = 0.007) and muscular strength (SMD = 0.17; *p* < 0.001). In a subgroup analysis that considered only lower-body exercises, there was no significant difference between caffeine and placebo for muscular endurance (SMD = 0.43; *p* = 0.092) or muscular strength (SMD = 0.16; *p* = 0.109). The main finding of this meta-analysis is that caffeine ingestion has a significant ergogenic effect on muscular endurance and muscular strength in women. The effects reported in this analysis are similar to those previously observed in men and suggest that women may use caffeine supplementation as an ergogenic aid for muscular performance. Future research is needed to explore the effects of caffeine on lower-body muscular endurance and muscular strength in this population.

## 1. Introduction

Caffeine is a highly popular ergogenic aid, often consumed by athletes and non-athletes alike [1,2]. A recent umbrella review of 21 meta-analyses concluded that caffeine ingestion is ergogenic for various components of exercise performance [3]. However, one limitation of the current body of evidence is that most primary studies are conducted among male participants. Specifically, the prevalence of primary studies with male-only samples ranged from 72% to 100% across the 21 meta-analyses included in the umbrella review [3]. This finding is in accord with a recent commentary that summarized data from 362 studies examining the effects of caffeine on exercise performance and found that only 13% of participants in the included studies were women [4]. 

Muscular endurance can be defined as “the ability of a muscle or muscle group to perform repeated contractions against a load for an extended period” [5]. In 2016, Polito et al. [6] published a meta-analysis that examined the effects of caffeine on muscular endurance. While caffeine ingestion was ergogenic for muscular endurance, out of the 16 analyzed studies, only one included women as the study participants. This study [7] included 15 resistance-trained women and explored the effects of caffeine in the dose of 6 mg/kg on muscular endurance using the bench press. In this study, there was no significant ergogenic effect of caffeine on muscular endurance, suggesting that the results observed among male participants may not necessarily be generalized to women. Therefore, if we examine the current meta-analytical data regarding the ergogenic effects of caffeine on muscular endurance, it is clear that these results are specific only to the male population. 

Besides muscular endurance, research has explored the effects of caffeine on muscular strength [8]. Muscular strength can be defined as “the capacity to exert force under a particular set of biomechanical conditions” [9]. A common test of muscular strength is the one-repetition maximum (1RM) test [8]. One meta-analysis reported that caffeine ingestion enhances 1RM strength [8]. The ergogenic effect of caffeine on 1RM strength was also found in a subgroup analysis that included only men [8]. However, in a subgroup analysis including only data collected in women, there was no significant difference between caffeine and placebo, possibly because only 3 studies (*n* = 33) were included [7,10,11]. 

In the last few years, researchers have noted that females comprise a smaller proportion of participants in sport and exercise research than males [12]. Accordingly, findings observed among male participants may not necessarily be generalized to the female population, highlighting the need for sex-specific research [13]. With regard to caffeine supplementation, one review suggested that caffeine intake produces a similar ergogenic effect on aerobic endurance in men and women but that the effects of caffeine on muscular endurance and muscular strength may be greater in men [14]. However, out of the 10 studies included in that review, only 3 and 2 studies focused on muscular strength and muscular endurance outcomes, respectively. In recent years, the interest in determining the ergogenic effect of caffeine in women has increased and several primary studies examined the effects of caffeine on muscular endurance and muscular strength in this population [15,16,17,18,19]. Accordingly, it is an opportune time to summarize their findings in a meta-analysis. Therefore, we aimed to conduct a meta-analysis exploring caffeine’s effects on muscular endurance and muscular strength in women. We hypothesized that caffeine ingestion would be ergogenic for muscular endurance and muscular strength.

## 2. Materials and Methods

### 2.1. Search Strategy

A total of five databases were searched to find studies that examined the effects of caffeine on muscular endurance and muscular strength in women. Specifically, we searched through Open Access Theses and Dissertations, PubMed/MEDLINE, Scopus, SPORTDiscus, and Web of Science. In all these databases, we applied the following search syntax: “caffeine” AND (“muscle strength” OR “muscular strength” OR “one-repetition maximum” OR “1RM” OR “resistance exercise” OR “resistance training” OR “muscle endurance” OR “muscular endurance”). After completing the primary search on 20 April 2021, secondary searches were carried out by examining papers that cited the included studies in Google Scholar and Scopus, as well as the included studies’ reference lists. The search was carried out independently by the two authors of the review. 

### 2.2. Inclusion Criteria

We included studies that satisfied the following criteria:

1. Published in English
2. Included women as study participants
3. Utilized a single or double-blind placebo-controlled crossover design
4. Examined the effects of isolated caffeine ingestion on 1RM strength and/or muscular endurance in an isotonic test involving “dynamic constant external resistance” (i.e., where the absolute external load is constant throughout the movement) [20].

Studies that included a mixed-sex sample were also considered for this review, as long as they presented individual group data for women.

### 2.3. Study Coding and Data Extraction

Data extraction was performed to obtain information about participants, intervention, comparisons, outcomes, and study findings. Specifically, the following data were extracted from the included studies:

1. Author(s), title, and year of publication
2. Participants’ characteristics (e.g., age, training status, habitual caffeine intake)
3. Caffeine supplementation protocol
4. Exercise test(s)
5. Main study findings

For one study [15] that presented the data in the form of figures, the Web Plot Digitizer software (https://apps.automeris.io/wpd/, accessed on 26 May 2021) was used to extract the necessary data. Standard errors (SEs) presented in one study [18] were converted to standard deviation (SD).

### 2.4. Methodological Quality

The methodological quality of the included studies was evaluated using the PEDro checklist [21]. The PEDro checklist has 11 items that assess different methodological aspects, such as inclusion criteria, randomization, allocation concealment, blinding, attrition, and data reporting. Each item is scored with “1” provided the criterion is satisfied; if the criterion is not satisfied, the item is scored with “0”. The first item on the PEDro checklist does not contribute to the total score, and therefore, the maximum possible number of points is 10. The studies were classified as excellent, good, fair, and poor methodological quality if they scored 9–10 points, 6–8 points, 4–5 points, and ≤3 points, respectively [22,23,24].

### 2.5. Statistical Analysis

This meta-analysis compared the effects of caffeine vs. placebo ingestion on muscular endurance and muscular strength. Muscular endurance and muscular strength performance data were converted to standardized mean differences (SMD) and are presented with their respective 95% confidence intervals (CI). The performance mean ± SD data (i.e., number of repetitions performed for muscular endurance or the weight lifted for muscular strength), total sample size, and inter-trial correlation were used to calculate SMDs, separately for each included study. The included studies did not present inter-trial correlation, and therefore, correlation values were estimated as suggested in the Cochrane Handbook [25]. Some studies evaluated performance under multiple caffeine conditions [10,15,18,19]. To account for these correlated effects within the same study, we first calculated SMDs and variances for each comparison and then used the average values in the main analysis. For example, one study [19] used a dose-response design and explored the effects of consuming either 3 or 6 mg/kg of caffeine. For this study, we calculated SMDs and variances individually for the comparison of placebo vs. 3 mg/kg of caffeine and the comparison of placebo vs. 6 mg/kg of caffeine. Then, we used the average SMD and variance values for the main analysis.

First, we performed an overall analysis with all articles to compare the effects of caffeine vs. placebo ingestion on muscular endurance (8 studies) and muscular strength (6 studies). Second, we performed subgroup meta-analyses to explore the effects of caffeine vs. placebo on lower-body and upper-body muscular endurance and muscular strength. All meta-analyses were performed using the random-effects model. SMD values were interpreted as: “small” (0.20–0.49), “medium” (0.50–0.79), and “large” (≥0.80), according to Cohen [26]. Heterogeneity was explored using the *I^2^* statistic. *I^2^* was interpreted as low (<50%), moderate (50–75%), and high heterogeneity (>75%). The statistical significance threshold was set at *p* < 0.05. All analyses were performed using the Comprehensive Meta-analysis software, version 2 (Biostat Inc., Englewood, NJ, USA).

## 3. Results

### 3.1. Search Results

**Figure 1:**
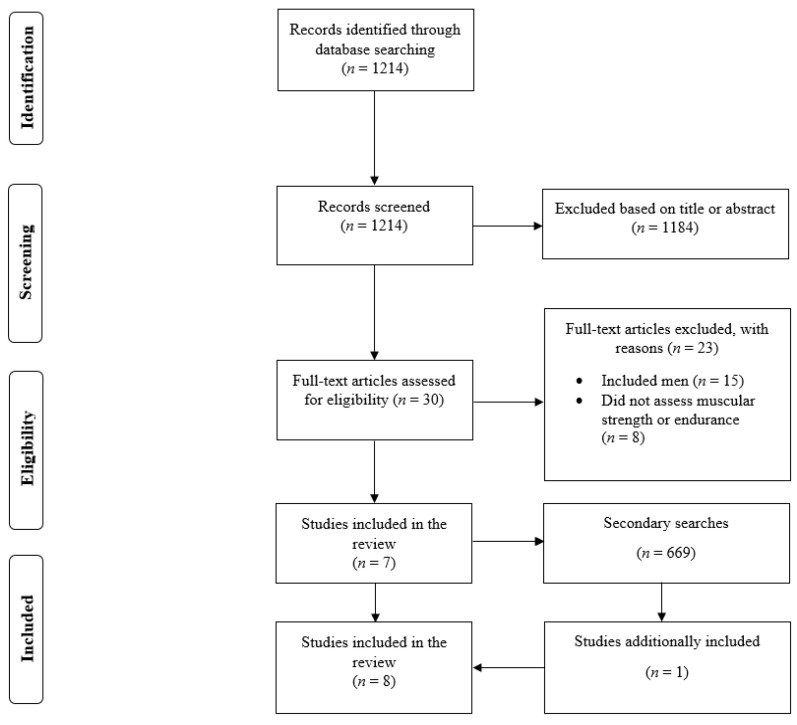
Flow diagram of the search process.

In the primary search, there was a total of 1214 results; 1184 documents were excluded after reading the title or abstract (Figure 1). After reading 30 full-text papers, 7 studies were found that satisfied the inclusion criteria [7,10,11,15,16,17,18]. In the secondary search, there were 669 search results, and one study was additionally included [19]. Therefore, we included a total of 8 studies in the present review [7,10,11,15,16,17,18,19].

### 3.2. Summary of Studies

All 8 studies (Table 1) explored the effects of caffeine on muscular endurance, while 6 studies examined the effects of caffeine on 1RM strength. Sample sizes in the included studies ranged from 8 to 29 participants. For studies that provided caffeine relative to body mass, the doses ranged from 2 to 6 mg/kg. One study [18] used two absolute doses (100 mg and 400 mg), which corresponded to relative doses of 1.4 and 5.6 mg/kg, respectively. Seven studies provided caffeine supplementation 60-min pre-exercise, and one study [15] used a protocol that involved caffeine ingestion 30-min pre-exercise. For upper-body exercises, studies used the bench press, bicep curl, lat-pull down, shoulder press, and row. For lower-body exercises, studies used the squat, leg press, or knee extension. Only one study [16] evaluated the effectiveness of the blinding and reported that 66% of the included participants were able to correctly identify the caffeine condition. None of the studies reported any commercial funding.

### 3.3. Methodological Quality

Five studies scored 9 points and were classified as being of excellent methodological quality (Table 2). Two studies scored 7 or 8 points and were classified as being of good methodological quality. One study scored 5 points and was classified as being of fair quality.

### 3.4. Meta-Analysis for Muscular Endurance

**Figure 2:**
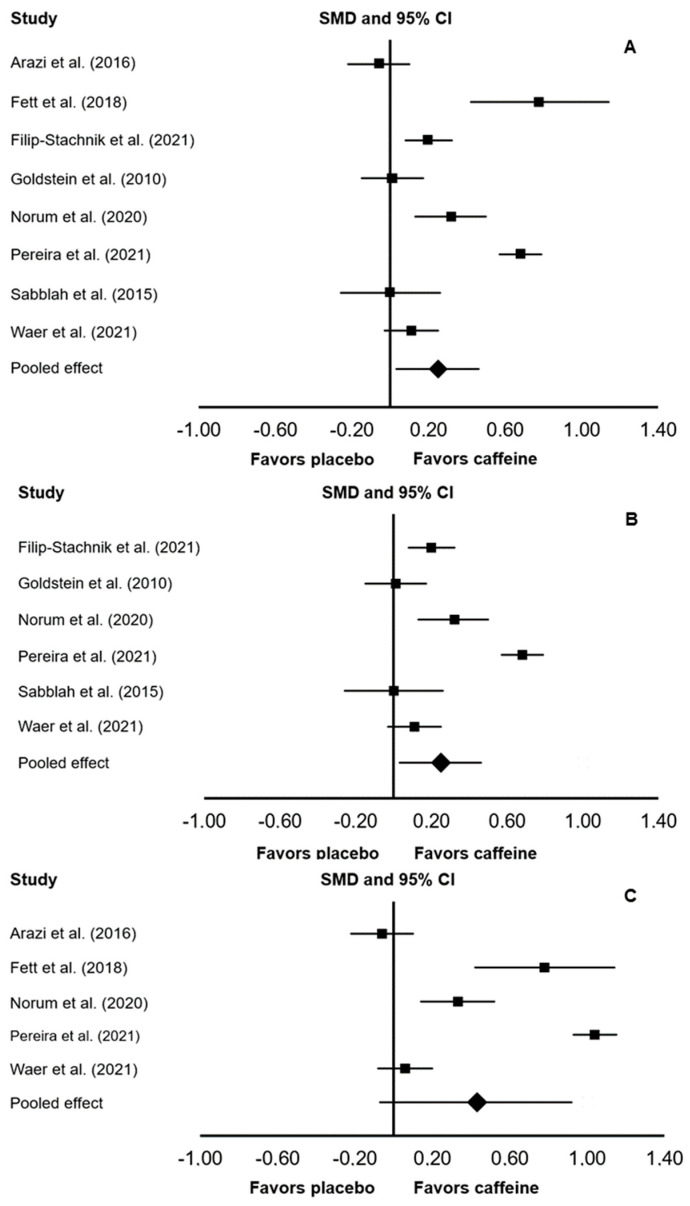
Forest plot presenting the results of the random-effects meta-analysis comparing the effects of placebo vs. caffeine on muscular endurance while considering all studies (A), only upper-body exercises (B), or only lower-body exercises (C). Data are reported as standardized mean differences (SMD) and 95% confidence intervals (CIs). The diamond at the bottom presents the overall effect. The plotted squares denote SMD, and the whiskers denote their 95% CIs.

In the main meta-analysis that considered data from 8 included studies, there was a significant ergogenic effect of caffeine on muscular endurance (SMD: 0.25; 95% CI: 0.03, 0.46; *p* = 0.027; *I*^2^ = 0%; Figure 2). In a subgroup analysis that considered only upper-body exercises, there was a significant ergogenic effect of caffeine on muscular endurance (SMD: 0.20; 95% CI: 0.05, 0.35; *p* = 0.007; *I*^2^ = 0%). In a subgroup analysis that considered only lower-body exercises, there was no significant difference between caffeine and placebo (SMD: 0.43; 95% CI: −0.07, 0.92; *p* = 0.092; *I*^2^ = 0%).

### 3.5. Meta-Analysis for Muscular Strength

**Figure 3:**
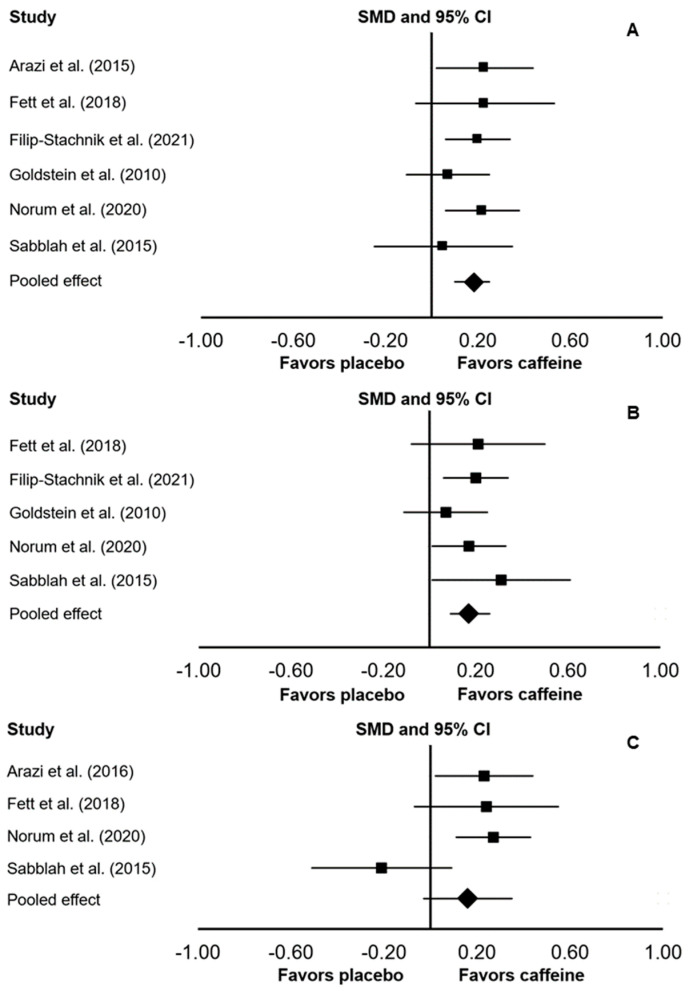
Forest plot presenting the results of the random-effects meta-analysis comparing the effects of placebo vs. caffeine on one-repetition maximum (1RM) muscular strength while considering all studies (A), only upper-body 1RM tests (B), or only lower-body 1RM tests (C). Data are reported as standardized mean differences (SMD) and 95% confidence intervals (CIs). The diamond at the bottom presents the overall effect. The plotted squares denote SMD, and the whiskers denote their 95% CIs.

In the main meta-analysis that considered data from 6 included studies, there was a significant ergogenic effect of caffeine on 1RM strength (SMD: 0.18; 95% CI: 0.10, 0.25; *p* < 0.001; *I*^2^ = 0%; Figure 3). In a subgroup analysis that considered only upper-body 1RM tests, there was a significant ergogenic effect of caffeine (SMD: 0.17; 95% CI: 0.09, 0.26; *p* < 0.001; *I*^2^ = 0%). In a subgroup analysis that considered only lower-body 1RM tests, there was no significant difference between caffeine and placebo (SMD: 0.16; 95% CI: −0.03, 0.35; *p* = 0.109; *I*^2^ = 0%).

## 4. Discussion

The main finding of this meta-analysis is that caffeine ingestion has a significant ergogenic effect on muscular endurance and muscular strength in women. In subgroup analyses, an ergogenic effect of caffeine was found on both muscular qualities only in the upper body. Nevertheless, it should be considered that the effects in the analysis for lower-body muscular endurance and muscular strength also favored the caffeine condition. From a practical perspective, it can be concluded that women interested in the acute enhancement of muscular endurance and muscular strength may consider caffeine supplementation pre-exercise. While the ergogenic effects were observed using caffeine doses from 2 to 6 mg/kg of caffeine, ingested from 30 to 60-min pre-exercise, future research is needed to establish an optimal protocol of caffeine supplementation for these outcomes. 

### 4.1. Effects of Caffeine on Muscular Endurance 

Based on our analysis, we conclude that caffeine ingestion is ergogenic for muscular endurance in women. In the investigations that used 1RM to normalize the load for the muscular endurance test, the load was between 40% and 80% of 1RM, suggesting that the utility of caffeine to enhance muscular endurance is obtained using moderate-to-high loads. While the majority of the studies used loads from 40% to 80% of 1RM, previous research in males also established an ergogenic effect of caffeine using lower loads (i.e., 30% of 1RM) or higher loads (i.e., 85% of 1RM), suggesting that the effects of caffeine on muscular endurance may not be load-dependent [28,29]. 

In the present meta-analysis, the pooled effect of caffeine on muscular endurance was 0.25, which is smaller than the effect size of 0.41 observed in a previous meta-analysis conducted among only male participants [6]. While this might suggest that the ergogenic effect of caffeine on muscular endurance is greater in men, we should also consider that the 95% CI in our analysis (i.e., 95% CI: 0.03, 0.46) largely overlapped with the 95% CI in the Polito et al.’s [6] meta-analysis (i.e., 95% CI: 0.31, 0.51). Therefore, the effects of caffeine on muscular endurance might be of similar magnitude in men and women.

The *I*^2^ values in all analyses were 0%, suggesting low heterogeneity. However, it should be considered that *I*^2^ estimates may be unreliable when working with less than ten included studies due to the lack of power [30]. Indeed, the effect sizes reported in individual studies varied. Specifically, two studies [15,17] reported effect sizes of 0.68 and 0.78, while the effects in all other studies [7,10,11,16,18,19] were smaller and ranged from –0.06 to 0.32. This variation in effects reported in the individual studies may be due to the utilized exercise tests. Specifically, studies that reported smaller effects utilized an exercise protocol involving only a single set performed to muscular failure. For example, in the study by Norum et al. [16], the participants were required to complete as many repetitions as possible in a single set with 60% of 1RM. Studies that reported larger effects used multiple sets performed to muscular failure. Fett et al. [15] used a drop-set protocol where the participants were required to reach muscular failure on three “drops” of weight—essentially, three sets with minimal inter-set rest. Pereira et al. [17] used a protocol where the participants performed three sets per exercise (all to failure) in five different exercises. Given that these two studies [15,17] reported effect sizes of 0.68 and 0.78, it might be that the ergogenic effect of caffeine on muscular endurance is larger in multiple set vs. single set protocols. This idea is also supported from a physiological perspective, given that caffeine ingestion has been shown to attenuate fatigue-induced loss of force production capability [31]. Future studies on this topic may consider using a multiple set exercise protocol (e.g., 3 sets per exercise) and then analyze the data from each set to directly establish the relationship between resistance exercise volume and caffeine’s ergogenic effect on muscular endurance. One study [32] adopted this approach, but the main outcome was barbell velocity during 4 sets of 8 repetitions while including a mixed-sex sample (i.e., 9 males and 3 females); therefore, future work on this topic is needed. 

### 4.2. Effects of Caffeine on Muscular Strength 

Our results corroborate previous meta-analytical data by Grgic et al. [8] that caffeine ingestion provides a significant ergogenic effect on 1RM strength. The data presented herein are novel, given that we observed this effect in women, while the previous findings are limited only to men. When discussing the effect size of caffeine on strength, it seems to be similar between men and women. Specifically, the effect of caffeine on 1RM strength in men amounted to 0.21 (95% CI: 0.03 to 0.38), which is nearly identical to the ergogenic effect observed in this meta-analysis (effect size: 0.18; 95% CI: 0.10, 0.25). Overall, this comparison suggests that the effectiveness of caffeine on muscular strength may be similar for both sexes. This notion is also supported by several studies that compared the effects of caffeine on men and women on aerobic and anaerobic performance and reported similar ergogenic effects in both sexes [33,34,35].

Due to factors such as muscle size, muscle activation, and motor unit recruitment, it has been previously suggested that caffeine’s ergogenic effect on muscular strength may be greater in the lower-body vs. upper-body [36,37,38,39,40]. However, we found an ergogenic effect of caffeine on upper-body 1RM strength, whereas no significant difference between caffeine and placebo was observed for the lower-body 1RM test. While this might suggest that the ergogenic effect of caffeine on 1RM strength is greater in the upper body, it should be considered that the effects also favored the caffeine condition for lower-body strength. Therefore, the lack of significance in this analysis might not indicate the absence of a true effect in the population, as it might be because only 4 studies [10,11,15,16] used lower-body 1RM tests. Therefore, future studies are still needed to explore the effects of caffeine on 1RM strength in different upper- and lower-body exercises in women.

### 4.3. Limitations 

The included studies were generally classified as good or excellent methodological quality on the PEDro checklist. However, one study [15] only scored 5 points and was classified as being of “fair” methodological quality. While this study was classified as “fair” on the PEDro checklist, due to its lower quality, the inclusion of this study might be considered a limitation of our review. Still, it should also be considered that this study had the smallest sample size (*n* = 8), which accounted only for ~6% of the pooled sample. Therefore, this study also had a low statistical weight in the analyses. There are some additional limitations observed among the included studies that should be considered for future research. For example, one study [11] used a single-blind design, which may be regarded as being of lower methodological quality than a double-blind design. While this study reported similar effects as all other included studies, future research should aim to use a double-blind design as this is considered the “gold standard” in sports nutrition [41].

Habitual caffeine intake among the participants included in the eight analyzed studies substantially varied. Specifically, some studies [10] included participants with a low habitual caffeine intake, while others included high habitual caffeine users [19]. This is important to consider as a potential limitation of the review, given that habitual caffeine intake may moderate the ergogenic effects of caffeine on exercise performance [42]. However, at least among the included studies, the ergogenic effects of caffeine did not seem to be influenced by habitual caffeine intake, given that performance-enhancing benefits were observed even among very high users of caffeine [19]. Still, future studies are needed to directly compare the effects of caffeine on muscular strength and muscular endurance among women with varying habitual caffeine intake. 

Recent data indicated that correct supplement identification impacts the outcome of an exercise task and may lead to bias in the results [43]. Out of the 8 included studies, only one study [16] evaluated the effectiveness of blinding and reported that 66% of participants correctly identified the caffeine condition. While relatively high, this study also conducted a between-group comparison of participants that identified caffeine vs. those that did not identify the caffeine condition and reported no significant differences between these two cohorts. This would suggest that the ergogenic effects observed in that study are not attributed to a placebo effect. Still, future studies should evaluate the effectiveness of blinding, given that the findings of this procedure may also impact data interpretation.

One aspect that should also be considered when conducting studies on the effects of caffeine on exercise performance in women is the phase of the menstrual cycle at which the testing is performed. It has been reported that caffeine metabolism varies in different phases of the menstrual cycle [44,45]. Only one included study [16] standardized for this variable, as all testing sessions were performed in the early follicular phase of the menstrual cycle. Given that only one study employed this procedure, this may be considered a limitation of our meta-analysis. However, recent studies observed similar exercise improvements following caffeine ingestion across all phases of the menstrual cycle [46,47]. Therefore, this limitation might not have confounded the results presented herein.

## 5. Conclusions

The main finding of this meta-analysis is that caffeine ingestion is ergogenic for muscular endurance and muscular strength in women. The effectiveness of caffeine on these muscular qualities was categorized as small, even though it was similar to the effects previously reported in male participants. In a subgroup analysis that considered only upper-body exercises, a significant ergogenic effect of caffeine was found for both muscular endurance and muscular strength. Even though the effects favoured caffeine, we did not find a significant difference between placebo and caffeine for lower-body muscular endurance and muscular strength. Therefore, future research is needed to explore the effects of caffeine on lower-body muscular endurance and muscular strength in this population. Overall, we conclude that women interested in the acute enhancement of muscular endurance and muscular strength may consider caffeine supplementation pre-exercise. 

## Figures and Tables

**Table 1:** Summary of studies included in the review.

Study	Participants	Habitual Caffeine Intake	Caffeine Supplementation	Muscular Strength Test	Muscular Endurance Test
Arazi et al. (2016)	10 teenage female karate athletes	<60 mg/day	2 or 5 mg/kg ingested 60 min pre-exercise	1RM leg press	60% of 1RM to muscular failure in the bench press (1 set)
Fett et al. (2018)	8 young resistance-trained women	Not reported	6 mg/kg ingested 30 min pre-exercise	1RM pull-down, hack squat, and bench press	Knee extension drop set using 100 kg, 80 kg, and 60 kg (1 set)
Filip-Stachnik et al. (2021)	21 resistance-trained women	5.8 ± 2.6 mg/kg/day	3 or 6 mg/kg ingested 60 min pre–exercise	1RM bench press	50% of 1RM to muscular failure in the bench press (1 set)
Goldstein et al. (2010)	15 young resistance-trained women	≤250 mg/day *n* = 8 ≥250 mg/day *n* = 7	6 mg/kg ingested 60 min pre-exercise	1RM bench press	60% of 1RM to muscular failure in the bench press (1 set)
Norum et al. (2020) *	15 young resistance-trained women	341 ± 184 mg/day	4 mg/kg ingested 60 min pre-exercise	1RM squat and bench press	60% of 1RM to muscular failure in the squat and bench press (1 set)
Pereira et al. (2021)	29 young resistance-trained women	Not reported	6 mg/kg ingested 60 min pre-exercise	n/a	Load of 10RM to muscular failure in the squat, leg press, bench press, shoulder press, and row (3 sets each exercise)
Sabblah et al. (2015)	8 young resistance-trained women	Not reported	5 mg/kg ingested 60 min pre-exercise	1RM squat and bench press	40% of 1RM to muscular failure in the bench press (1 set)
Waer et al. (2021)	19 healthy middle-aged women	<200 mg/day	100 or 400 mg ingested 60 min pre-exercise	n/a	As many repetitions as possible in 30 s in the squat (no external load) and biceps curl with 2.27 kg (1 set)

RM: repetition maximum; * study controlled for menstrual cycle phase; according to the Filip et al. [27] habitual use of caffeine can be classified as follows: “naïve consumer”—<25 mg/day; “low consumer”—25 mg/day to 0.99 mg/kg/day; “mild consumer”—1.00–2.99 mg/kg/day; “moderate consumer”—3.00–5.99 mg/kg/day; “high consumer”—6.00–8.99 mg/kg/day; “very high consumer”—>9.00 mg/kg/day.

**Table 2:** Results from the PEDro checklist.

Study	Item 1	Item 2	Item 3	Item 4	Item 5	Item 6	Item 7	Item 8	Item 9	Item 10	Item 11	TS
Arazi et al. (2016)	Yes	Yes	No	Yes	Yes	Yes	Yes	Yes	Yes	Yes	Yes	9
Fett et al. (2018)	Yes	No	No	Yes	Yes	No	No	No	Yes	Yes	Yes	5
Filip-Stachnik et al. (2021)	Yes	Yes	No	Yes	Yes	Yes	Yes	Yes	Yes	Yes	Yes	9
Goldstein et al. (2010)	Yes	Yes	No	Yes	Yes	Yes	Yes	Yes	Yes	Yes	Yes	9
Norum et al. (2020)	Yes	Yes	No	Yes	Yes	Yes	Yes	No	Yes	Yes	Yes	8
Pereira et al. (2020)	Yes	Yes	No	Yes	Yes	Yes	Yes	Yes	Yes	Yes	Yes	9
Sabblah et al. (2015)	No	Yes	No	Yes	Yes	No	No	Yes	Yes	Yes	Yes	7
Waer et al. (2021)	Yes	Yes	No	Yes	Yes	Yes	Yes	Yes	Yes	Yes	Yes	9

No: criterion is not satisfied; Yes: criterion is satisfied; excellent quality (9–10 points); good quality (6–8 points); fair quality (4–5 points); poor quality (less than 3 points); first item is not included in the summary score; TS: total score.

## Data Availability

The data used for analysis are available on request from the corresponding author.
